# COVID-19 Prevention and Control Measures in Workplace Settings: A Rapid Review and Meta-Analysis

**DOI:** 10.3390/ijerph18157847

**Published:** 2021-07-24

**Authors:** Carolyn Ingram, Vicky Downey, Mark Roe, Yanbing Chen, Mary Archibald, Kadri-Ann Kallas, Jaspal Kumar, Peter Naughton, Cyril Onwuelazu Uteh, Alejandro Rojas-Chaves, Shibu Shrestha, Shiraz Syed, Fionn Cléirigh Büttner, Conor Buggy, Carla Perrotta

**Affiliations:** School of Public Health, Physiotherapy, and Sports Science, University College Dublin, D04 V1W8 Dublin, Ireland; vicky.downey@ucd.ie (V.D.); mark.roe@ucd.ie (M.R.); yanbing.chen@ucd.ie (Y.C.); mary.archibald@ucdconnect.ie (M.A.); kadri-ann.kallas@ucdconnect.ie (K.-A.K.); jaspal.kumar@ucdconnect.ie (J.K.); peter.naughton@ucdconnect.ie (P.N.); cyril.onwuelazuuteh@ucdconnect.ie (C.O.U.); alejandro.rojas-chaves@ucdconnect.ie (A.R.-C.); shibu.shrestha@ucdconnect.ie (S.S.); shiraz.syed@ucd.ie (S.S.); fionn.cleirigh-buttner@ucdconnect.ie (F.C.B.); conor.buggy@ucd.ie (C.B.)

**Keywords:** occupational health and safety, infection prevention, COVID-19, control measures, workers, review

## Abstract

Workplaces can be high-risk environments for SARS-CoV-2 outbreaks and subsequent community transmission. Identifying, understanding, and implementing effective workplace SARS-CoV-2 infection prevention and control (IPC) measures is critical to protect workers, their families, and communities. A rapid review and meta-analysis were conducted to synthesize evidence assessing the effectiveness of COVID-19 IPC measures implemented in global workplace settings through April 2021. Medline, Embase, PubMed, and Cochrane Library were searched for studies that quantitatively assessed the effectiveness of workplace COVID-19 IPC measures. The included studies comprised varying empirical designs and occupational settings. Measures of interest included surveillance measures, outbreak investigations, environmental adjustments, personal protective equipment (PPE), changes in work arrangements, and worker education. Sixty-one studies from healthcare, nursing home, meatpacking, manufacturing, and office settings were included, accounting for ~280,000 employees based in Europe, Asia, and North America. Meta-analyses showed that combined IPC measures resulted in lower employee COVID-19 positivity rates (0.2% positivity; 95% CI 0–0.4%) than single measures such as asymptomatic PCR testing (1.7%; 95% CI 0.9–2.9%) and universal masking (24%; 95% CI 3.4–55.5%). Modelling studies showed that combinations of (i) timely and widespread contact tracing and case isolation, (ii) facilitating smaller worker cohorts, and (iii) effective use of PPE can reduce workplace transmission. Comprehensive COVID-19 IPC measures incorporating swift contact tracing and case isolation, PPE, and facility zoning can effectively prevent workplace outbreaks. Masking alone should not be considered sufficient protection from SARS-CoV-2 outbreaks in the workplace.

## 1. Introduction

The novel SARS-CoV-2 is a respiratory pathogen causing COVID-19 [1]. Transmission is associated with exposure to droplets, fomites, and aerosols, particularly in crowded or confined spaces [2,3]. Asymptomatic infections occur in 17–21% of cases [4]. These dynamics can result in COVID-19 outbreaks and superspreading events (SSEs) that lead to changes in the community reproductive number [5].

Workplaces are common settings for explosive infectious disease outbreaks due to transmission between employees and their close contacts in respective households and communities [6]. As employees have a legal right to a safe workplace, employers must create safe working conditions [7]. However, in the absence of evidence on COVID-19 infection prevention and control (IPC) measures, employers have relied on two methods. First, many employers have applied the precautionary principle of a “better safe than sorry” approach comprising a bundle of measures for all employees [8]. Second, employers have sought direction from guidelines on preventing transmission of other respiratory pathogens such as influenza and severe acute respiratory syndrome (SARS) that have been modified for COVID-19 [9]. These approaches have had consequences, namely global shortages of personal protective equipment (PPE) and increased unemployment due to workplace closures [10]. Now, more than 15 months since SARS-CoV-2 emerged, it is crucial to identify measures that prevent and control pathogen transmission in workplace settings. Effective IPC measures can guide future efforts to respond to global health emergencies by protecting susceptible employees and citizens [11]. Therefore, this rapid review aims to investigate the effectiveness of measures to prevent and control COVID-19 outbreaks in workplace settings. Specifically, this study seeks to (1) map the IPC measures investigated in included studies and (2) assess COVID-19 positivity estimates associated with IPC measures implemented in workplace settings.

## 2. Methods

Given the urgency to identify evidence-based IPC measures for preventing COVID-19 outbreaks in workplace settings, a rapid review was completed.

### 2.1. Research Questions

The Problem/Population, Intervention, Comparison, and Outcome (PICO) framework was used to formulate research questions facilitating a precise search for occupational IPC measures (I/C) that reduce COVID-19 transmission (O) in the workplace environment (P) [12]. The following research questions were defined:

1. What COVID-19 IPC measures are used in workplace settings?
2. What IPC measures reduce COVID-19 infections in the workplace?

### 2.2. Workplace COVID-19 IPC Measures

Prior to study selection and analysis, COVID-19 IPC measures were broadly categorized according to World Health Organization (WHO) guidelines for health and safety in the workplace [13]. Categories were defined as:

1. *Surveillance measures*—COVID-19 symptom monitoring, strategies to screen or test individuals, such as symptomatic or asymptomatic testing;
2. *Outbreak investigations and response*—contact tracing and testing of close contacts, quarantine of potentially infected individuals or groups, self-isolation of confirmed cases;
3. *PPE*—masks, full PPE (i.e., masks, goggles, gloves, work clothes) in medium/high-risk jobs;
4. *Environmental adjustments*—improving airflow and ventilation, adding physical barriers to help employees avoid physical contact, environmental cleaning;
5. *Education initiatives*—training on IPC measures, communication and signage, ongoing education and support;
6. *Changes in work arrangements*—social distancing, facility zoning, entrance restrictions, changes in assignments for high-risk workers (i.e., individuals with medical conditions, pregnant women, over-60 population), facility shutdown, paid sick leave;
7. *Combined measures*—approaches that combine measures from two or more categories.

### 2.3. Defining Effectiveness 

Articles that formally assessed or modelled whether the implemented workplace measures prevented and/or controlled workplace transmission of SARS-CoV-2 were considered in this review. IPC measures that result in (i) lower numbers of confirmed cases among all employees tested following the intervention (i.e., COVID-19 positivity), (ii) lower secondary attack rates among (non-)household contacts, (iii) higher percentage reductions in effective reproductive number (*R*_eff_), or (iv) percentage of COVID-19 cases prevented, may generally be considered more effective. However, in the case of surveillance and contact tracing, a high positivity percentage may be considered more effective, given that more cases are being successfully identified. We take care over the course of this review to consider contextual variability and endeavour not to overstate effective and ineffective interventions.

### 2.4. Search Strategy

Publications in any language were identified on four electronic databases: Medline, Embase, PubMed, and Cochrane Library. The following search terms were used: SARS-CoV-2 OR “severe acute respiratory syndrome coronavirus” OR “SARS” AND (workplace OR employers OR “healthcare workers” OR “nursing homes” OR “residential care” OR “meat factories” OR “factories” OR industry OR occupation OR “occupational health” OR “creche” OR “childcare facilities” OR work*).

### 2.5. Study Selection (Identification, Screening, and Inclusion)

Studies that were conducted in any workplace and geographical setting were included if they met at least one of the following criteria, by undertaking: (1) surveillance of a population over a specified time period, (2) an outbreak investigation, (3) an assessment of a COVID-19 IPC measure or policy, or (4) mathematical modelling to estimate effectiveness.

Studies that met the following criteria were excluded: (1) mental health outcomes, (2) modelling studies with assumptions of effectiveness (e.g., effectiveness of IPC measures as modelling assumption(s) rather than outcome(s) to be predicted), (3) systematic reviews, and (4) qualitative investigations, including commentaries and editorials. Though included in our initial search strategy, studies related to SARS-COV or MERS-CoV were eventually excluded as they were less relevant to the novel SARS-CoV-2/COVID-19. 

Identified studies were imported into Covidence systematic review software (Veritas Health Innovation, Melbourne, Australia). Covidence automatically removed duplicate studies. To rapidly screen studies, 20 postgraduate researchers trained in public health and medicine were enlisted. In pairs, researchers screened the title and abstract of imported studies. The same process was repeated for full text review, with eligible articles subsequently included for data extraction.

### 2.6. Data Extraction

For each included article, two researchers independently extracted data into structured Excel sheets. Any discrepancies in extracted data were identified by the principal investigator (PI) and resolved by discussion between extractors. Data on the country of study conduct, study design, workplace setting, population investigated, IPC measure(s) implemented, outcome(s) measured, study start and end dates, and study duration were extracted from the included studies. Studies were selected for quantitative synthesis if they (1) had complete denominators, (2) assessed a similar intervention to at least one other study, (3) included a comparable outcome measure, and (4) were deemed sufficiently homogeneous in clinical and methodological characteristics to permit meta-analysis. For quantitatively aggregable studies, additional information was compiled on the national COVID-19 positivity estimate(s) during the study period, and the trajectory of the national pandemic wave during that time (e.g., acceleration, deceleration, peak) according to the Johns Hopkins Coronavirus Resource Center [14]. For included studies that implemented combined measures, the total number of interventions implemented was also recorded.

### 2.7. Quality Assessment 

Given the diversity of included study designs and interventions, risk of bias could not be assessed using a single, existing tool. An adapted checklist modelled after Gulumian et al. [15] was used to score longitudinal studies based on experimental study (yes/no), total study population reported (yes/no), gold-standard PCR testing used (yes/no), and intervention follow-up time reported (yes/no). Scores ranged from 1 (lowest) to 4 (highest). Cross-sectional studies automatically received a quality score of 1 due to their low quality of evidence [16].

The Checklist for critical Appraisal and data extraction for systematic Reviews of prediction Modelling Studies (CHARMS) was used to assess the quality of modelling studies. Modelling studies were assessed for risk of bias according to source of data, outcome to be predicted, candidate predictors, sample size, missing data, model development, model performance, model evaluation, results, interpretation, and discussion. Quality assessment was completed by six reviewers and discrepancies were solved mutually between reviewers.

### 2.8. Statistical Analysis

Random-effects meta-analyses were performed using the DerSimonian–Laird method to estimate the pooled effect of IPC measures implemented in two or more studies. Because we assumed, prior to model selection, that aggregated studies would not share a common effect size (due to between-study variation in study design, setting, and intervention), random-effects models were considered more appropriate than fixed-effects models [17]. Study weights were assigned using the inverse of each study’s total variance (i.e., by combining within- and between-study variation). An arcsine-based transformation was used to stabilize the variance of each study’s proportion estimate through a conventional, two-step meta-analytic approach [18]. First, each study’s COVID-19 positivity estimate was transformed using the Freeman–Tukey double arcsine transformation to approximate a normal distribution required for meta-analysis. Then, meta-analytic results on the transformed scale were back-transformed to interpret pooled estimates and 95% confidence intervals (95% CI) [19]. The Freeman–Tukey double arcsine transformation method was chosen as it can account for studies with zero or one effect size [19]. Cochran’s Q test was performed to estimate whether total variation was statistically different compared to expected variation when assuming that all aggregated studies share a common, underlying positivity estimate. We also calculated a Tau-squared (T^2^) parameter and I^2^ statistic to estimate between-study variance and the proportion of total variation that is due to real differences between studies’ positivity estimates, respectively. We selected I^2^ > 75% to define “high” heterogeneity. Outlying effect sizes were identified by screening for externally studentized residuals (Z > 2) and excluded if they exerted considerable influence on summary effect size [20].

When 10 or more studies assessed similar interventions using the same outcome variable, univariate meta-regression analysis was performed to examine the influence of other factors on intervention effectiveness. The following predetermined factors were studied: study region, duration of intervention, community transmission rate during intervention period, community pandemic wave interval, implementation of specific interventions (asymptomatic PCR testing, facility zoning, education, environmental cleaning, PPE, syndromic surveillance, contact tracing), and the total number of interventions implemented. To account for multiple comparisons, a Bonferroni correction was applied, and the significance threshold was set at *p* < 0.01. The significance threshold for all other tests was set at *p* < 0.05. 

When study numbers allowed (*n* ≥ 10), publication bias was tested for using Egger’s test. Sensitivity analysis was performed by repeating meta-analyses while excluding low-quality studies (adapted checklist quality score < 2). All statistical analyses were completed using R version 4.0.2 (R Foundation for Statistical Computing, Vienna, Austria). Meta-analyses and meta-regressions were performed using the R packages meta 4.18–0 and metafor 2.4–0.

## 3. Results

### 3.1. Study Characteristics

**Figure 1:**
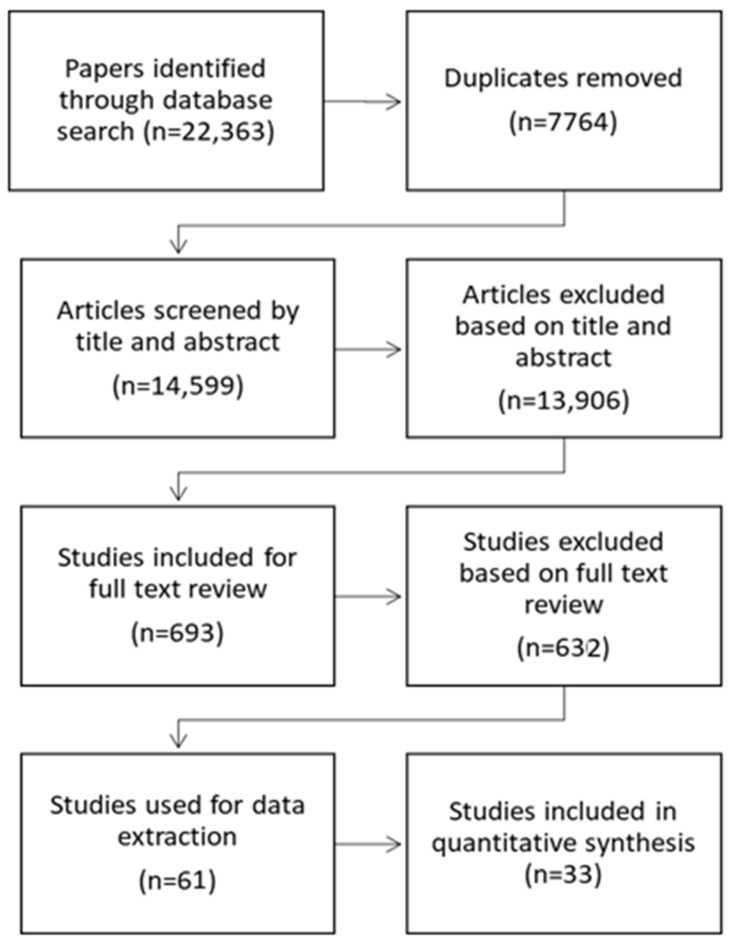
PRISMA flowchart.

A total of 22,363 studies, published through 19 April 2021, were imported for screening. Following duplicate removal, title and abstract screening, and full-text screening, 61 met all inclusion criteria and were included in the review (Figure 1).

Included studies (k = 61) varied in study design, comprising 6 cross-sectional studies [21,22,23,24,25,26], 7 case-control series [27,28], 22 prospective cohort studies [29,30,31,32,33,34,35,36,37,38,39,40,41,42,43,44,45,46,47,48,49,50], 1 longitudinal cohort study [51], 7 retrospective cohort studies [52,53,54,55,56,57,58], 7 prospective observational studies (i.e., observing the number of new cases in a facility after the implementation of IPC measures) [59,60,61,62,63,64,65], 1 retrospective observational study [66], 3 outbreak investigation reports [67,68,69], 1 post hoc analysis [70], 1 sequential follow-up study [71], 1 short-term prospective survey [72], 2 surveillance studies [73,74], and 7 mathematical modelling studies [75,76,77,78,79,80,81]. All studies were conducted before COVID-19 vaccinations became available in December 2020.

The number of participants in each study ranged from 18 to 53,000, equating to 276,350 total participants. The studies yielded from North America, Asia, and Europe. Fifteen studies were performed in the United States (USA) [28,36,38,41,45,49,51,52,55,69,72,78,79,80,81]. Other included studies were performed in India [32], France [72], Canada [29,63], Italy [22,42,44,57,60,62,74], the United Kingdom (UK) [35,40,50,53,58,65,75,76], Belgium [21,39], Korea [30,31,54,56], Taiwan [73], Germany [33,37,59,67,68,71] China [48], Finland [25], Spain [26], Japan [43], Singapore [47,61,64], Vietnam [34,70], and Malaysia [46]. The remaining studies included were performed across multicentre international settings [24,27,77].

Hospitals and healthcare centres (*n* = 45) were the most common workplace settings in the included studies, followed by nursing homes (*n* = 11), offices (*n* = 2), manufacturing facilities (*n* = 1), meat factories (*n* = 1), and modelling of general workplaces (*n* = 1). Table 1, Table 2 and Table 3 present an overview of the study characteristics and IPC measures from healthcare studies (Table 1), nursing home studies (Table 2), and other workplaces (Table 3).

### 3.2. Study Quality

Of the 61 included studies, 48 were of longitudinal design (i.e., researchers examined the same group of workers over a period of time). The amended checklist assessment for longitudinal studies showed that only one of these was experimental [70]. Two of the 48 longitudinal studies did not report a complete denominator [41,67], whereas 19 out of 48 did not specify intervention follow-up time. No study attained the highest overall quality score of 4. Twenty-three studies had a quality score of 3, and 19 had a quality score of 2. Studies with a quality score of 1 (k = 12), including cross-sectional studies, were considered low-quality evidence (Table 1 and Table 2). Evaluations of mathematical modelling studies using the CHARMS checklist identified low risk of bias in the seven modelling studies included in this review. Detailed quality assessment findings for all studies are provided in Supplementary Materials.

### 3.3. Effectiveness of Workplace COVID-19 IPC Measures

COVID-19 IPC measures implemented by studies included in the rapid review comprised six categories: (i) surveillance, (ii) outbreak investigation and response, (iii) PPE, (iv) changes in work arrangements, (v) worker education, and (vi) combined measures. No studies assessing the effectiveness of environmental adjustments were identified. Measures tested as part of mathematical modelling studies were considered separately due to their hypothetical nature. Table 4, Table 5 and Table 6 map the array of single, combined, and modelled measures implemented by category and the studies that assessed their effectiveness. 

All 61 studies contained a statistical measure of effectiveness for one or more COVID-19 IPC measures. More than half of the studies (34/61) used COVID-19 positivity rates to assess effectiveness between 4 and 270 days following the implementation of IPC measures. Except in the case of single testing and contact tracing measures, lower positivity was considered more effective. For test and trace measures, higher positivity was considered more effective as it meant COVID-19 cases were successfully being captured. Other outcome types included attack rates, mean reduction in R_eff_, odds ratios (OR), relative risk (RR), and hazards ratios of COVID-19 infection. The median intervention duration was 41 days (min 1–max 300). Thirty-three studies contained results that were amenable to meta-analyses of proportion estimates, stratified by intervention type (Table 7). Note that no studies falling into the ”changes in work arrangements” and ”worker education” intervention categories met inclusion criteria for formal quantitative synthesis.

#### 3.3.1. Surveillance Measures

In terms of single IPC measures implemented, COVID-19 surveillance was identified most often in the literature (k = 17), particularly asymptomatic RT-PCR testing of employees [28,30,35,38,39,40,49,57,63,65,74]. Asymptomatic testing was carried out in a number of ways: universally [30,35,38,39,40,49,57,63,65,74], on a voluntary basis [37], following an outbreak [21,36], according to environmental surface testing [24] or contact tracing [35,40], and during point prevalence surveys in hospitals and nursing homes [36,69].

Pooled COVID-19 positivity estimates amongst employees who underwent universal, asymptomatic RT-PCR testing (25,023 HCWs from nine studies; 54 office employees from one study) was 1.7% (95% CI = 0.9–2.9) (Table 7). Egger’s regression asymmetry test was non-significant (*p* = 0.93), indicating the potential absence of publication bias. To explore heterogeneity between asymptomatic testing interventions (I^2^ = 96%), univariate meta-regression was conducted; however, none of the pre-identified potential moderating factors—that is, study region and duration, rates of community transmission, and community pandemic wave interval—were significantly associated with greater COVID-19 positivity or reduction in heterogeneity.

#### 3.3.2. Outbreak Investigation and Response

Nine studies assessed the effectiveness of outbreak investigations involving contact tracing and ongoing surveillance [31,35,40,47,54,60,64,73,74]. Pooled COVID-19 positivity estimates from these studies amongst 6599 total HCWs was 3.5% (95% CI = 0.8–7.9). Positivity estimates from individual studies varied according to interventions implemented. In-depth syndromic surveillance and outbreak investigations [47,73] led to less than 0.5% hospital-acquired infections amongst the HCWs tested. Contact tracing paired with mass screening (*n* = 5942 HCWs) [74] and testing of close contacts (*n* = 1730 close contacts) [60] identified higher rates of infection (10% COVID-19 positivity). Two studies carried out contact tracing and symptomatic testing of UK hospital workers in April 2020, detecting 1.9% (5/267) and 7.7% positivity (4/52) estimates, respectively [35,40]. A study that performed contact tracing in South Korea, and demonstrated the highest positivity estimate amongst studies in that meta-analysis, identified that 17.2% of hospital staff exposed to known COVID-19 cases (15/87) either had, or subsequently, tested positive for COVID-19 [54]. Notably, community transmission was low during the study period (i.e., ~2% COVID-19 positivity).

#### 3.3.3. PPE

The effectiveness of PPE in reducing workplace COVID-19 transmission was assessed in 12 studies [26,27,32,33,41,43,45,52,58,66,70]. In the healthcare setting, three studies assessed the effectiveness of general PPE [26,33,43], and three assessed universal masking policies [41,45,55]. One study looked at the effectiveness of using N95 respirators in the hospital setting compared to surgical masks [27]. Studies also assessed the effectiveness of cloth masks compared to medical masks [70], of having adequate PPE supply [66], of homemade PPE for laboratory workers in India [32], and of how eye protection compares to face protection [58].

Pooled positivity estimates were high following single universal masking interventions implemented in the two PPE studies with results amenable to meta-analysis (Table 7). Of 11,648 total HCWs tested for COVID-19 two-to-three weeks following universal masking interventions, 24% tested positive for COVID-19 (95% CI 3.4–55.5). Individually, universal masking studies reported 11.5% (1129/9850) [55] and 39.5% (725/1834) post-intervention positivity [45], accounting for 3.2% and 4.4% reductions in COVID-19 positivity, respectively.

#### 3.3.4. Combined Measures

Fifteen studies used multiple IPC measures at once, as displayed in Table 5. The most widely used intervention component was PPE (universal masking, full PPE in high-risk healthcare settings) [22,34,44,46,47,48,62,68,71]. Kong and Yan added a unique component to their intervention: inventory monitoring protocols to ensure adequate PPE supply at all times [48]. Other widely used measures in combined interventions were universal, asymptomatic testing [29,42,48,56,62,71], symptomatic testing [29,46,47,67,68], and social distancing [22,47,48,67,68]. Five studies conducted employee education and/or training [22,34,44,48,67]. Kabesch et al. implemented on-site visits by hygiene experts as part of their intervention in a German hospital, similar to those whose effectiveness was assessed by Lipsitz et al. in the USA [51,67]. Several combined interventions also included contact tracing [44,46].

**Figure 2:**
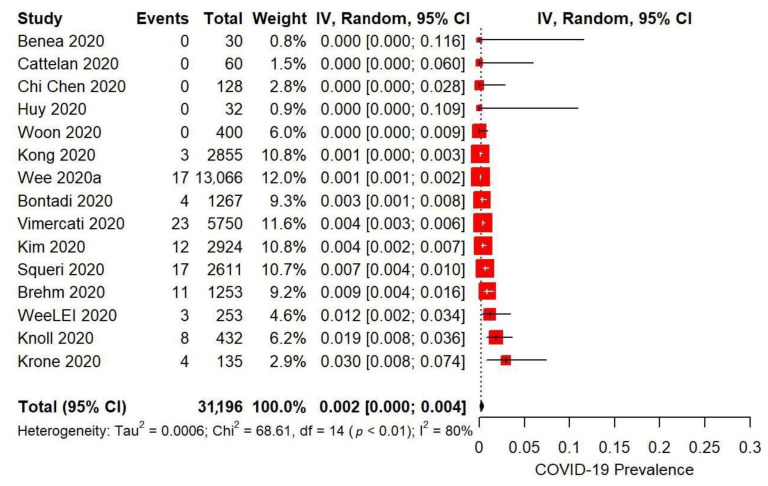
Meta-analysis of COVID-19 positivity rates in workplaces (14 hospitals and 1 nursing home) following the implementation of combined IPC measures between January and September 2020. Exact interventions implemented by each study are detailed in Table 5. Note that two studies ([64,73]) were classified under the ”Outbreak Investigations and Response” category in Table 4. We have chosen to include them in the Combined Measures meta-analysis due to their comprehensive nature.

Studies that used combined measures had a lower pooled COVID-19 positivity estimate at the end of their intervention period than studies that implemented single measures (Table 7, Figure 2).

Across the 15 studies that implemented combined IPC measures and could be included in meta-analysis, 0.2% (95% CI 0.0–0.4) of 31 196 healthcare and nursing home workers tested positive for COVID-19. Univariable meta-regression analysis was conducted to assess for pre-determined moderators that might explain between-study heterogeneity (I^2^ = 80%) (Table 8). Studies conducted in Europe had higher positivity than studies conducted in Asian countries (QM (df = 2) = 7.14, *p* = 0.03), with study region explaining ~43% of effect size heterogeneity. Studies conducted in communities with over 5% positivity during the intervention period were not significantly more likely to experience higher COVID-19 positivity estimates (QM (df = 1) = 3.39, *p* = 0.07), whereas implementing a higher number of interventions resulted in significantly lower COVID-19 positivity estimates (QM (df = 1) = 6.10, *p* = 0.01). Other than asymptomatic testing (QM (df = 1) = 4.96, *p* = 0.02), no single intervention type resulted in significantly lower COVID-19 transmission in studies that implemented it compared to those that did not. Study duration was also not significantly associated with COVID-19 positivity (QM (df = 1) = 43.96, *p* = 0.21).

Egger’s regression asymmetry test indicated potential for publication bias amongst the 15 studies that implemented combined measures (*p* = 0.04) (Table 4); however, sensitivity analyses excluding lower-quality evidence (Quality Score < 2) did not affect pooled effect sizes or meta-regression results.

#### 3.3.5. Key Findings from Modelling Studies

Modelling studies provided additional support for the effectiveness of combined IPC measures versus single interventions, particularly when implemented early (Table 9). Models showed that random, asymptomatic testing in settings with low to moderate SARS-CoV-2 transmission rates did little to stop outbreaks [77,80], whereas asymptomatic outbreak testing effectively prevented workplace infections [81]. Testing alone was not enough to reduce R_0_ below 1 [76] but effectively reduced transmission when paired with high-quality infection control practices [81]. Timely and widespread contact tracing (<24 h contact after the first case identified, ~80% coverage) [75,76], swift self-isolation of cases [76], and effective use of PPE [77] were reported to slow and/or prevent SARS-CoV-2 transmission. Other effective IPC measures included creating smaller worker/patient cohorts in hospitals [77], and restricting patient contact for high-risk HCWs over the age of 60 [78].

#### 3.3.6. Key Findings from Studies Not Included in Quantitative Synthesis

Due to the high level of heterogeneity in terms of study type, interventions assessed, and outcome measured, results from 28 studies could not be included in formal quantitative synthesis. From these, several studies exemplary (quality score > 1) that demonstrate the effectiveness of diverse interventions have been selected, key findings from which are summarized in Table 10.

#### 3.3.7. Summary of Findings

The review identified 61 articles that implemented and assessed COVID-19 IPC measures in the workplace. The studies showed that universal asymptomatic RT-PCR testing yielded low employee positivity rates, indicating few cases identified at potentially high cost in moments of reduced community transmission. Asymptomatic testing more effectively captured cases when implemented following facility outbreaks or environmental monitoring; however, studies generally concluded that all testing should be combined with high-quality workplace infection control practices. Staff compartmentalization within zones and/or cohorts (worker bubbles), for example, was identified as an effective way to prevent workplace transmission. Universal masking, though a critical component of most initiatives to protect workers, proved inadequate in reducing workplace transmission when implemented alone. Masking was more effective when combined with physical barriers. While studies mentioned the added value of environmental adjustments and worker education for maximizing masking efficiency, there was a gap in quantitative evidence supporting this. Results from contact tracing interventions varied widely, indicating the potential moderating role of community transmission rates and/or other contextual factors in contact tracing effectiveness. Nevertheless, ongoing syndromic surveillance and outbreak investigations tended towards lower post-intervention COVID-19 positivity estimates than once-off contact tracing and testing initiatives. Mathematical modelling demonstrated the role that contact tracing coverage and timeliness, and added physical distancing measures, could play in maximizing the effectiveness of test-and-trace initiatives.

Meta-analyses using random-effects models supported these findings, highlighting lower COVID-19 positivity estimates in workplace settings that implemented combined measures compared with settings that applied single measures. Though different regional and workplace contexts prevent the identification of a cure-all combination of measures, PPE, timely and thorough outbreak investigations, syndromic surveillance and testing, and staff compartmentalization within zones emerge as important considerations.

## 4. Discussion

Over one year into the COVID-19 pandemic, workplace settings remain a high-risk environment for SARS-CoV-2 outbreaks, presenting great risk to the health and well-being of employees, their families, and surrounding communities. Due to the rapidly changing nature of COVID-19 and related management, control, and prevention guidelines, employers have struggled to implement timely and effective COVID-19 protective measures in the workplace [82]. To develop a greater understanding, this rapid review of the literature was designed to compile evidence on COVID-19 IPC measures implemented in global workplace settings through April 2021. Specifically, this review (1) mapped existing measures and (2) assessed their effectiveness.

Despite high levels of heterogeneity in study type, region, setting, and outcomes measured, there was a consensus in the literature on the increased effectiveness of combined versus single measures, thereby providing evidence in support of layered mitigation strategies recommended by national and international health authorities [13,83]. Unsurprisingly, meta-regression revealed a positive association between the total number of interventions implemented and reduced employee COVID-19 positivity estimates. However, this begs the question of cost-effectiveness since workplaces, particularly in low-income developing countries, may lack resources to implement exhaustive measures. Fortunately, Juneau and colleagues considered the question of cost-effectiveness in their own systematic review and identified swift contact tracing and case isolation, surveillance networks, PPE, and early vaccination when possible as the most cost-effective combination of interventions, particularly when adopted early [84]. Workplace closures have been reported as effective but costly [84].

Appropriate combinations of measures may also vary depending on workplace context. Healthcare settings, for example, must consider which type of PPE is most effective (low-quality evidence suggests that N95 respirators should be used during aerosol generating procedures (AGP) instead of surgical masks) [27,85], or the possibility of restricting the age of workers with direct patient contact [77]. Food and manufacturing facilities have other, unique considerations such as how environmental conditions, sick leave policies, and access to health insurance for employees affect worker vulnerability to COVID-19. Unfortunately, no evidence was identified on how these factors related to COVID-19 transmission in the meatpacking and manufacturing facilities included in this review.

Other significant research gaps emerged. Most studies assessed the effectiveness of IPC measures in hospital and nursing home settings, demonstrating the extent to which healthcare facilities and staff have been disproportionately impacted by the COVID-19 pandemic. However, few if any studies assessed IPC measures in manufacturing, industrial, essential retail, and public service settings, which have continued to provide essential goods and services to the public throughout the global pandemic. The geographical spread of included studies was concentrated in Europe, Central Asia, and North America, revealing a gap in emerging literature from South America, Southern Asia, Oceania, and Africa. Research on COVID-19 occupational safety measures is critical to protect essential workers in epicentres such as Brazil, Argentina, Colombia, India, and Peru, where case numbers rank amongst the highest in the world [14]. Conversely, Australia and New Zealand have been remarkably adept at preventing local COVID-19 outbreaks [14]; however, no literature was identified detailing how their workers were effectively protected.

Though several studies implemented education initiatives as part of combined interventions, few assessed the effectiveness of worker education specifically. Research has shown that HCWs can feel overwhelmed by constantly changing IPC guidelines, that adherence to guidelines is influenced by levels of support and communication from management, that training is most effective when mandatory, and that there is a need for training on the infection itself and proper PPE use [9]. In meatpacking plants, home to internationally diverse worker cohorts, language barriers have been identified as a primary challenge in deploying COVID-19 IPC strategies but the effectiveness of implemented strategies such as multilingual signage and mass-communication apps remains unassessed [86]. Moreover, this review found little evidence on how cultural factors may be influencing workplace viral transmission and/or interacting with COVID-19 IPC measures. Such findings underline the pressing need for research on how tailored education initiatives can help protect workers.

Our rapid review identified another gap in evidence surrounding effective contact tracing methods. While included studies mentioned contact tracing frequency and coverage, none discussed methods for ensuring adequate public engagement despite research identifying privacy concerns, mistrust, unmet needs for information, and digital challenges as potential barriers to engagement and subsequent contact tracing effectiveness [87]. Finally, given strong evidence that SARS-CoV-2 spreads by airborne transmission [3], this review revealed a need for experimental research on how indoor environmental adjustments relate to COVID-19 outbreaks and superspreading events. While the WHO has released guidelines for improved workplace ventilation, our lack of findings on indoor air adjustments supports claims that precise ventilation or air-purification regimes to improve workplace safety remain unknown [88]. Countries have begun implementing promising, low-cost strategies for reducing workplace risk through improved indoor air quality. In Belgium, for example, COVID-19 ventilation rules requiring carbon dioxide monitors to be on public display have been imposed, allowing workers and members of the public to determine when air quality reaches unsafe levels [89]. Yet, experts note that a lack of studies addressing CO_2_ monitoring as a public health tool during the pandemic leads to uncertainty surrounding optimal CO_2_ levels for COVID-19 conditions [90]. This type of evidence will be of great value to the indoor workforce moving forward. Scientists and public health agencies recommend free and low-cost options for preventing viral transmission inside such as opening windows, using window fans, turning off demand-controlled ventilation controls, and repositioning supply/exhaust diffusers [83]. Workplaces should strongly consider adding these adjustments to the layered IPC approach identified as effective by this review.

### 4.1. Completeness and Applicability of Evidence

Concerning the compiled evidence base, most included studies were observational in nature, which may impact the strength of findings and recommendations. The necessity of responding to a global emergency meant that some researchers had to document workplace transmission as it unfolded, or to assess outbreaks retrospectively. These assessments provide important insight on how to protect workers from COVID-19; however, they do not allow for strong conclusions on the effectiveness of particular IPC measures. Experimental studies provide higher-quality evidence but, as they are difficult to design and carry out in emergency settings, only one was identified for inclusion in this rapid review. This review also included mathematical modelling studies that, while providing support for the effectiveness of timely and comprehensive workplace interventions, may vary in terms of real-world applicability. Results from this review are therefore not intended to guide clinical practice, but to enhance the understanding of what IPC measures have been implemented, and/or implemented effectively, in workplaces thus far.

### 4.2. Limitations and Strengths

Our rapid review and meta-analysis presented several limitations and challenges. First, due to the time frame of the grant through which this rapid review was funded (Science Foundation Ireland Grant 20/COV/8539) and limited availability of graduate student volunteers, we were required to begin data extraction as soon as our protocol was complete. This disqualified the review from subsequent submission to the online PROSPERO database. Additional challenges were encountered during formal analysis. Nearly half of included studies could not be included in meta-analyses due to variability of outcome variables and/or incomplete denominators. This resulted in several small pools of comparable studies (k = 2–3), potentially impacting the robustness of meta-analytic results. Though we sought to test community COVID-19 transmission rates as a potential moderating factor using meta-regression, many studies reported long intervention periods and/or imprecise study locations, making it difficult to identify precise community transmission rates. This may have contributed to why community transmission was only marginally associated with COVID-19 positivity in the workplace. Many potential moderators of the relationship between IPC measures and pooled positivity estimates were deemed to be non-significant using meta-regression. However, our inability to detect statistically significant associations for many of these pre-determined moderators may not be due to a non-existent or meaningless effect but rather a lack of statistical power (due to too few studies) to detect a small but meaningful difference between subgroups. Additionally, few studies reported changes in COVID-19 outcomes over time, making it difficult to account for temporal dynamics of workplace transmission when assessing intervention effectiveness. Finally, this rapid review contains a potentially unrepresentative sample of global workplaces as most included studies yielded from healthcare, nursing home, European, Asian, and North American settings. Results should therefore be interpreted with caution when applied to other professional, geographical, and/or cultural contexts.

Despite these limitations, this rapid review makes several important contributions to global understanding of COVID-19 in workplace settings. To our knowledge, this is the first study to map all COVID-19 IPC measures implemented and assessed in workplace settings. Most included studies were performed in healthcare settings, which has important implications for other workplace settings. By demonstrating that high-risk healthcare settings were able to effectively contain and/or prevent workplace COVID-19 outbreaks, this rapid review demonstrates the feasibility of lower-risk workplace settings remaining open at minimized risk to employees and the wider community. As our study demonstrates, this will require combinations of surveillance, swift contact tracing and case isolation, facility zoning, and universal masking. This study shows that masking alone should not be considered sufficient protection against workplace outbreaks. Despite widespread vaccination programs, COVID-19 case numbers remain high worldwide and workplace safety is of critical importance. Our findings can help to protect workers in countries where businesses are beginning to reopen and public interactions increasing; in developing countries where infections remain uncontrolled; and in future settings when other respiratory diseases with airborne transmission threaten workplace safety.

### 4.3. Implications for Research

This rapid review highlights the extent to which workplaces including hospitals, nursing homes and, to a lesser extent, manufacturing, meatpacking, and office settings are at increased risk for SARS-CoV-2 outbreaks and in need of timely and effective infection prevention and control measures. Moving forward, experimental studies that address identified gaps in the workplace COVID-19 IPC literature base are of pressing importance. This includes research on effective IPC education initiatives, on ways to maximize public engagement in contact tracing, and on how precise environmental conditions relate to COVID-19 transmission in the workplace. While this rapid review identified a large body of evidence on how to protect healthcare and social care workers, further research should consider ways to better support essential workers in retail, education, construction, transportation, and manufacturing settings. High-quality evidence on effective workplace COVID-19 IPC measures in under-studied and highly positive regions such as Central-South America and South Asia is also necessary to ensure the protection of *all* workers, not just those in wealthy countries.

## 5. Conclusions

Workplaces remain common settings for infectious disease outbreaks and superspreading events. Though many national and global health authorities provide recommendations for workplace health and safety, there has been a reported absence of evidence on precise COVID-19 IPC regimes to make workplaces safe. By conducting a synthesis of the evidence base on workplace COVID-19 IPC measures and their effectiveness, our results can inform guidelines on how to better protect workers from COVID-19 and future infectious disease outbreaks. Moving forward, timely and comprehensive IPC measures should be favoured in workplace settings. Though exact combinations of measures may vary depending on professional, geographical, or cultural context, our rapid review identified swift and thorough contact tracing and case isolation, effective PPE, syndromic surveillance and testing, and staff zoning and/or cohorts as important considerations. These measures should be paired with improved building ventilation and indoor air quality. Governments should prioritize funding for these initiatives, particularly for small businesses who may lack financial resources for adequate IPC programs. Masking alone should not be considered sufficient protection from SARS-CoV-2 outbreaks in the workplace. Our findings indicate that applying timely and comprehensive infection prevention and control measures can allow workers to safely remain in or return to the workplace in the context of COVID-19 and at the outset of future epidemics.

## Figures and Tables

**Table 1:** Study characteristics and COVID-19 IPC measures implemented in hospital and other healthcare settings.

Scheme	Design	Country	Setting	Population	Quality Score/4 ^a^	Infection Prevention and Control Measures
[32]	Prospective Cohort Study	India	Hospital	Residents (*n* = 5), lab technicians (*n* = 10), nursing orderlies (*n* = 3)	2	In-house, homemade tools for standard operating procedures: face masks, OT gowns
[29]	Prospective Cohort Study	Canada	Hospital and nursing home residences	HCWs^+^ (*n* = 30)	3	Home-based 7-day control strategy for exposed HCWs, asymptomatic RT-PCR testing
[57]	Retrospective Cohort Study	Italy	Teaching hospital	Patients and HCWs (*n* = 103)	2	Nasal swab qPCR and IgG/IgM antibodies testing
[62]	Prospective Observational Study	Italy	Hospital	HCWs (*n* = 7595)	3	Separated and dedicated COVID areas, multiple hand hygiene installations, PPE, training protocols, implementation of surveillance system
[30]	Prospective Cohort Study	Korea	Hospital	HCWs and patients (*n* = 142)	3	Universal screening programme
[80]	Modelling Study	USA	Healthcare facilities	Residents and HCWs (*n* = 100)	LRB ^b^	Routine asymptomatic PCR testing
[31]	Prospective Cohort Study	Korea	Hospital setting	HCWs (*n* = 317)	2	Contact tracing
[63]	Prospective Observational study	Canada	Tertiary care centre	HCWs (cohort 1: *n* = 1669, cohort 2: 4107, cohort 3: *n* = 1597)	3	Symptomatic/asymptomatic nasopharyngeal swab PCR testing
[65]	Prospective Observational Study	UK	Hospital setting	Staff (*n* = 10,034)	3	Naso-/oropharyngeal swab and/or immunoassay IgG testing; contact tracing
[75]	Mathematical Modelling	UK	*n*/A	Not reported	LRB ^b^	Estimate of PCR test sensitivity, sensitivity and specificity of IgG antibody test, positive predictive value of a positive antibody test
[73]	Surveillance Study	Taiwan	Hospital	HCWs (*n* = 374)	3	Online body temperature surveillance, outbreak investigation and management, advising HCWs not to travel
[34]	Prospective Descriptive Study	Vietnam	Hospital laboratory Setting	Staff members (*n* = 32)	2	Risk assessment and management, laboratory training program, self-reporting and electronic reporting of COVID-19 symptoms, PPE stock monitoring system
[33]	Prospective Seroprevalence Study	Germany	Hospital setting	Clinical and non-clinical MRI staffs (*n* = 6305), and medical students (*n* = 1699)	3	PPE; PCR testing for SARS-CoV-2, anti-SARS-CoV-2 IgG and IgM testing
[79]	Modelling	USA	Hospital	HCW’s (*n* = 1350)	LRB ^b^	Nasopharyngeal samples
[35]	Prospective Cohort Study	UK	Teaching hospital	HCWs (*n* = 360)	3	Symptomatic/asymptomatic HCW screening
[67]	Outbreak Investigation Report	Germany	Maternity and Perinatal centre	Not reported	2	Extensive testing; universal face masks; central monitoring of sick leaves; measures to ensure social distancing; continuous on-site visits by hygiene experts and staff training
[36]	Prospective Cohort Study	California, USA	Skilled nursing facility	Hospital staff and residents (*n* = 725)	2	Targeted testing: point prevalence surveys.
[56]	Retrospective Cohort Study	Korea	Hospital setting	Patients and HCWs (*n* = 2924)	2	Nasopharyngeal and oropharyngeal swabs, surveillance of people with contact history with confirmed COVID-19 patients.
[68]	Outbreak Investigation Report	Germany	Tertiary university hospital	HCWs (*n* = 432)	3	Quarantine of positive HCWs, containment measures including surgical masks; physical distancing, and systematic testing.
[48]	Cohort Study	China	Tertiary hospital	Patients (*n* = 1860)	2	Hospital layout adjustments, specialized training, pre-testing and triage, environmental cleansing, PPE
[23]	Cross-Sectional	France	Hospital	HCW’s (*n* = 314)	1	Nasal swab testing, self-isolation, and masks
[39]	Prospective Cohort Study	Belgium	Hospital	HCWs (*n* = 699)	2	SARS-CoV-2 RNA and anti-SARS-CoV-2 IgG antibodies testing
[27]	Case-Control study	International	*n*/A (online survey)	HCWs (*n* = 1130)	1	Use of respirators for aerosol generating procedures (AGP); PPE use and training
[25]	Cross-Sectional	Finland	Tertiary hospitals	HCWs (*n* = 1072)	1	Social distance of 1 m
[44]	Prospective Cohort Study	Italy	Hospital	HCWs (*n* = 5750)	2	Contact tracing, reinforced hygiene practices, PPE, education, and signage
[70]	Post hoc Analysis of a Randomized Controlled Trial	Vietnam	Hospital	HCWs (*n* = 607)	3	Washing method for cloth masks
[37]	Prospective Cohort Study	Germany	Hospital	Hospital staff (*n* = 1185)	3	Low-threshold SARS-CoV-2 testing facility
[26]	Cross-Sectional Study	Spain	Hospital setting	Hospital Workers (*n* = 2963)	2	Use of PPE
[74]	Surveillance Study	Italy	Hospital setting	Staff and residents under contract working (*n* = 5942)	3	Mass screening (oropharyngeal and nasopharyngeal swabs) with/without contact tracing
[77]	Mathematical Modelling Studies	International	Healthcare setting	HCWs (*n* = 224)	LRB ^b^	Surveillance
[78]	Modelling Study	USA	Hospital setting	Hospital Workers (*n* = 53,000, number of hospital workers in the US)	LRB ^b^	Use of PPE in all healthcare workers. Use of PPE only in high-risk workers. Restricting age of workers < 60 y; restricting age of workers < 50 y
[40]	Prospective Cohort Study	UK	Teaching hospital	HCWs (*n* = 1032), symptomatic HCW’s (*n* = 169), symptomatic household contacts (*n* = 52)	3	Asymptomatic screening using real-time RT-PCR Symptomatic screening using real-time RT-PCR Symptomatic screening of household contacts
[38]	Prospective Cohort Study	USA	Electrophysiology unit	Staff (*n* = 912) and patients (*n* = 758)	2	Universal asymptomatic testing for patients, caregivers, staff, and emergency medical service staff
[41]	Prospective Cohort Study	USA	2 Community hospitals	HCWs (*n* = 21,014)	1	Universal masking
[42]	Prospective Cohort Study	Italy	Hospital	HCWs (*n*-2611)	1	PPE and sanitation guidelines implemented, epidemiological investigation and contact tracing of high-risk HCWs, symptomatic swab testing
[60]	Prospective Observational Study	Italy	2 Large hospitals	HCWs (6800)	3	Contact tracing and testing of close contacts; random testing
[43]	Prospective Cohort Study	Japan	Hospital setting	HCWs (*n* = 49)	1	PPE (90% compliance)
[71]	Short-Term Prospective Study	Germany	Tertiary care centre	Staff (*n* = 1253)	3	Multimodal infection control: strict barrier nursing of known COVID-19 patients, including full PPE, visitor restrictions, universal face masks, universal RT-PCR admission screening of patients
[54]	Retrospective Cohort Study	Korea	Healthcare setting	Hospital staff (*n* = 87) and patients (*n* = 224)	3	Outbreak investigation surveillance
[55]	Retrospective Cohort Study	USA	Hospitals	HCWs (*n* = 9850)	3	Universal masking for HCWs
[45]	Prospective Cohort Study	USA	Hospital	HCWs (*n* = 832)	3	Universal face mask policy
[47]	Prospective Cohort Study	Singapore	Hospital	HCWs (*n* = 1642)	3	Enforcing reporting of HCWs with acute respiratory illness (ARI) to staff clinic for monitoring; ongoing syndromic surveillance; outbreak investigation and management
[61]	Prospective Observational Study	Singapore	Hospital	HCWs (*n* = 13,066)	3	Multi-tiered infection control strategy: improved patient segregation and distancing, heightened infection prevention and control measures including universal masking, testing of all symptomatic patients
[64]	Prospective Observational Study	Singapore	Hospital	Staff (*n* = 253)	3	Contact tracing; 14-day phone surveillance and 28-day follow-up of close contacts; testing of symptomatic contacts
[46]	Prospective Cohort Study	Malaysia	Hospital	HCWs (*n* = 400)	2	Full PPE, which includes an N95 mask, an isolation gown, gloves, eye protection and a head cover when providing care to patients under investigation or confirmed COVID-19 patients, and anti-SARS-CoV-2 antibodies serological tests

^a^ Studies scored from 1 to 4 according to experimental design, total study population reported, PCR testing used, and follow-up time reported. Cross-sectional studies automatically scored 1 due to their high risk of bias. ^b^ LRB = Low risk of bias according to Checklist for Critical Appraisal and Data Extraction for Systematic Reviews of Prediction Modelling Studies (CHARMS) + HCW = Healthcare worker.

**Table 2:** Study characteristics and COVID-19 IPC measures implemented in nursing home settings.

Study Reference	Design	Country	Population	Quality Score/4 ^a^	Infection Prevention and Control Measures
[66]	Retrospective Observational Study	France	Staff (*n* = 360) and residents (*n* = 930)	2	Nursing home has enough masks for all residents and staff
[58]	Retrospective Cohort Study	UK	Nurses, care workers and non-care workers	2	Increased PPE: face masks, eye protection
[21]	Cross-Sectional Study	Belgium	Staff (*n* = 93) and residents (*n* = 119)	1	Anti-SARS-CoV-2 antibody testing in addition to RT-PCR testing
[81]	Modelling	USA	Residents and staff (*n* = 215)	LRB ^b^	Serial testing of asymptomatic persons in response to an outbreak; serial testing of asymptomatic healthcare personnel in the absence of known cases
[59]	Prospective Observational Study	Germany	Staff (*n* = 135) and Residents (*n* = 160)	2	General screening and cohort isolation
[53]	Retrospective Cohort Study	UK	Care home staff (*n* = 474)	2	Nasal swab testing; working in multiple vs. single care home
[51]	Longitudinal Cohort Study	Massachusetts, USA	Care homes (*n* = 360)	2	6-part intervention: 28-item checklist, payment incentive, on-site and virtual infection control consultation, weekly webinars, continuous question and answer communication, PPE-staffing-testing resources
[72]	Short-Term Prospective Study	France	Long-term care facilities (*n* = 124)	2	Staff compartmentalization within zones; self-assessment scale of the quality of the “barrier” measures
[50]	Cohort Study	UK	Staff (*n* = 320); residents (*n* = 349)	1	Implementation of a negative pressure isolation space
[28]	Case-Series Study	Washington, USA	Staff (*n* = 62) and residents (*n* = 80)	1	Enhanced hygiene practices were implemented
[69]	Outbreak Investigation	USA	Nursing facilities (*n* = 26)	3	Universal asymptomatic testing for patients, caregivers, and staff

^a^ Studies scored from 1 to 4 according to experimental design, total study population reported, PCR testing used, and follow-up time reported. Cross-sectional studies automatically scored 1 due to their high risk of bias. ^b^ LRB = Low risk of bias according to Checklist for Critical Appraisal and Data Extraction for Systematic Reviews of Prediction Modelling Studies (CHARMS).

**Table 3:** Study characteristics and COVID-19 IPC measures implemented in other workplace settings.

Study Reference	Design	Country	Setting	Population	Quality Score/4 ^a^	Infection Prevention and Control Measures
[22]	Cross-Sectional Study	Italy	Manufacturing facility	Employees (*n* = 1267)	1	Social distancing, individual hygiene rules, PPE, cleaning and sanitizing of environments, information, and training of workers
[49]	Prospective Cohort Study	USA	Offices	Employees (*n* = 27), household (*n* = 27), students, and volunteers	2	Nasal swabs, RT-qPCR measuring antibodies concentration by ELISA
[76]	Modelling Study	UK	General population	(*n* = 40,162)	LRB ^b^	Physical distancing, isolation, tracing, and testing
[24]	Cross-Sectional: Point Prevalence	Belgium, Spain, Italy, France, USA, UK	Offices and industrial buildings	Workplaces (*n* = 411 for 1st week/*n* = 424 for 2nd week)	1	Environmental monitoring
[52]	Retrospective Cohort Study	USA	Meatpacking facility	Employees (*n* = 1000)	1	PPE and physical barriers

^a^ Studies scored from 1 to 4 according to experimental design, total study population reported, PCR testing used, and follow-up time reported. Cross-sectional studies automatically scored 1 due to their high risk of bias. ^b^ LRB = Low risk of bias according to Checklist for Critical Appraisal and Data Extraction for Systematic Reviews of Prediction Modelling Studies (CHARMS).

**Table 4:** Map of single workplace COVID-19 measures implemented by category.

Preventive Measures Category	Study Reference
Surveillance	
Asymptomatic PCR testing	[28,35,38,39,49,57,63,65]
Symptomatic PCR testing	[23,35,40,63]
Symptomatic PCR testing of household contacts	[35,40]
RT-PCR testing of staff after environmental monitoring	[24]
Asymptomatic IgG/IgM immunoassay testing	[57,65]
Asymptomatic IgG/IgM immunoassay testing following an outbreak	[21]
Asymptomatic RT-PCR testing following an outbreak	[21]
Point prevalence surveys	[36,69]
Low-threshold SARS-CoV-2 testing facility	[37]
Outbreak Investigations and Response	
Syndromic surveillance, outbreak investigations	[47,73]
Contact tracing	[31]
Mass screening, contact tracing	[74]
Contact tracing, testing of close contacts	[60]
Contact tracing, 14-day phone surveillance, 28-day follow-up of close contacts	[64]
Asymptomatic RT-PCR prior to patient surgery, contact tracing of exposed HCWS	[54]
PPE	
Cloth masks compared to medical masks	[70]
Universal masking	[41,45,55]
Homemade tools for standard operating procedures	[32]
High PPE compliance	[26,33,43]
Adequate PPE supply	[66]
Masks with and without physical barriers	[52]
Respirators used instead of surgical masks	[27]
Eye protection and face protection	[58]
Education	
On-site and virtual infection control consultations	[51]
Changes in work arrangements	
Staff compartmentalization within zones	[72]
Negative pressure isolation space	[50]
Restricted worker mobility between facilities	[53]
Social distancing compliance	[25]

**Table 5:** Map of combined workplace COVID-19 measures implemented.

Combined Preventive Measures.	Study Reference
Hospital layout adjustments, training, pre-testing and triage, environmental cleansing, PPE	[48]
Standard operating procedure, staff training, symptom reporting, enhanced cleaning, inventory monitoring protocols	[34]
Social distancing, universal masking, testing of all symptomatic patients	[61]
Home-based 7-day infection control strategy for exposed HCWs—symptomatic, asymptomatic RT-PCR testing	[29]
General screening and cohort isolation	[42]
PPE and sanitation guidelines implemented, epidemiological investigation and contact tracing of high-risk HCWs, symptomatic swab testing	[46]
Integrated infection control strategy: zoning, PPE, mass surveillance	[62]
PPE, visitor restrictions, universal face masks, universal RT-PCR patient admission screening	[71]
Hospital shut down, universal testing of all inpatients, medical staff, and employees	[56]
Systematic testing, social distancing, monitoring of sick leaves, on-site visits by hygiene experts, staff training, direct communication of all measures to personnel and patients	[67]
Social distancing, surgical masks, systematic testing	[68]
Regulation of access to the company, social distancing, hygiene and PPE, cleaning and sanitizing of environments, worker education	[22]
Contact tracing, reinforced hygiene practices, PPE, education, and signage	[44]

**Table 6:** Map of modelled workplace COVID-19 measures.

Modelled Preventive Measures.	Study Reference
Variations in employee testing frequency (daily, weekly, bi-weekly, monthly)	[80]
Variations in testing frequency; outbreak vs. non-outbreak testing	[81]
Testing and symptomatic isolation; regular screening of high-risk groups; close contact quarantine	[75]
Non-adaptive combinatorial matrices used for group testing	[79]
Self-isolation and variations in contact tracing methods; mass testing	[76]
Variations in PPE use; worker age restrictions	[78]
Variations in PPE efficacy and testing frequency	[77]

**Table 7:** Meta-analysis of COVID-19 positivity rates according to the IPC measures implemented.

Intervention	No. of Studies	*n*	Pooled Positivity Rate (%) ^a,b^	95% CI	Q	I^2^	T^2^	*p*-Value	Egger’s Test ^c^	Egger’s Test *p*-Value
**Asymptomatic RT-PCR ^d^**	10	25077	1.7 ^e^	0.9, 2.9	202.32	96%	0.0025	<0.01	0.09	0.93
**Surveillance and Contact tracing**	9	6599	3.5 ^e^	0.8, 7.9	391.59	98%	0.0191	<0.01	*n*/A	*n*/A
**Universal Masking of Employees**	2	11684	24.0 ^f^	3.4, 55.5	692.34	100%	0.0559	<0.01	*n*/A	*n*/A
**Combined measures (>2 intervention categories)**	15	31196	0.2 ^f^	0.0, 0.4	68.61	80%	0.0006	<0.01	2.24	0.04

^a^ Inverse variance method. ^b^ Freeman–Tukey double arcsine transformation. ^c^ Minimum of 10 studies or greater. ^d^ We chose to focus on asymptomatic RT-PCR because positivity rates from other testing interventions—symptomatic testing (naturally high positivity; value is in subsequent contact tracing and case isolations) and antibodies testing (does not capture active COVID-19 infections) —less accurately depict IPC effectiveness. ^e^ High positivity = generally more effective (cases effectively captured). ^f^ Low positivity = generally more effective (virus effectively prevented/controlled).

**Table 8:** Univariable meta-regression results for 15 studies that implemented combined IPC measures between January and September 2020.

Factor	QM (df)	R^2^	Beta coefficient (99% CI)	Standard Error	*p*-Value ^a^
**Region**	7.137 (2)	43%			
Europe vs. Asia			0.048 (0.0368, 0.0371)	0.010	0.008 **
North America vs. Asia			0.041 (0.0402, 0.0425)	0.093	0.223
North America vs. Europe			0.004 (0.003,0.006)	0.093	0.963
**Intervention Duration (Days)**	43.96 (1)	12%	−0.0002 (−0.0002, −0.0002)	0.0001	0.214
**Community COVID-19 positivity (Under 5% vs. Over 5%)**	3.389 (1)	0%	−0.036 (−0.0358, −0.0353)	0.019	0.066
**Pandemic Wave Interval**	0.820 (2)	0%			
Deceleration vs. Acceleration			0.028 (0.027,0.028)	0.033	0.394
Peak vs. Acceleration			0.005 (0.004,0.005)	0.023	0.848
**Specific Intervention Implemented (Yes vs. No)**					
Asymptomatic RT-PCR testing	4.961 (1)	27%	0.040 (0.0394,0.0399)	0.018	0.023 *
Facility Zoning	0.040 (1)	0%	0.004(0.0038,0.0044)	0.021	0.842
Employee Education	1.610 (1)	0%	−0.026 (−0.026, −0.026)	0.020	0.205
Environmental Cleaning	3.733 (1)	0%	−0.038 (−0.038, −0.038)	0.020	0.053
PPE	2.133 (1)	14%	−0.025 (−0.025, −0.025)	0.017	0.144
Syndromic Surveillance	2.210 (1)	16%	−0.026 (−0.027, −0.026)	0.018	0.137
Contact Tracing	0.330 (1)	0%	−0.012 (−0.012, −0.012)	0.021	0.566
**Total Interventions Implemented**	6.102 (1)	22%	−0.0109 (−0.0110, −0.0108)	0.004	0.014 *

* *p* < 0.05 ** *p* < 0.001. ^a^ Significance level set at *p* < 0.01 after application of Bonferroni correction.

**Table 9:** Key findings from studies that modelled the effectiveness of workplace COVID-19 surveillance and combined IPC measures.

Study	Risk of Bias	Key Findings
[80]	Low	Asymptomatic testing frequency in a healthcare environment depends on baseline R0 In an environment with R_0_ = 2.5, testing would have to occur almost every other day to bring R0 below 1 If assuming R0 = 1.5, testing weekly would suffice
[81]	Low	Asymptomatic outbreak testing in nursing homes could prevent 54% (weekly testing with 48-h test turnaround) to 92% (daily testing with immediate results and 50% relative sensitivity) of SARS-CoV-2 infections Adding non-outbreak testing could prevent up to an additional 8% of SARS-CoV-2 infections All testing should be combined with high-quality infection control practices
[75]	Low	The effectiveness of test and trace depends strongly on coverage and the timeliness of contact tracing Molecular testing can play an important role in prevention of SARS-CoV-2 transmission, especially among healthcare workers and other high-risk groups, but no single testing strategy will reduce *R0* below 1 at low levels of population immunity
[79]	Low	Non-adaptive combinatorial group testing works well at low SARS-CoV-2 prevalence levels; however, performance decreases as prevalence levels increase
[76]	Low	A high proportion of cases would need to self-isolate and a high proportion of their contacts to be successfully traced to ensure an effective reproduction number lower than 1 in the absence of other measures. Self-isolation and contact tracing measures would be more likely to achieve control of SARS-CoV-2 transmission if combined with moderate physical distancing measures
[78]	Low	Availability of PPE for high-risk HCWs could prevent nearly half of hospital acquired COVID-19 infections Restricting hospital workers above the age of 60 from direct patient care could reduce infections by up to 96%
[77]	Low	Effective use of PPE by both HCWs and patients could prevent overwhelmed healthcare systems, while random testing of apparently asymptomatic/pre-symptomatic individuals on a weekly basis was less effective Creating smaller patient/HCW interaction subcohorts can provide additional resilience to outbreak development

**Table 10:** Short description of the effectiveness of selected interventions.

Study	Intervention Category/Setting	Findings	Conclusions
[24]	Surveillance/Hospital	OR calculation for locations with PCR or antibody positives (2400 environmental swabs) vs. locations without positives (3000 environmental swabs) reveals that locations with coronavirus-positive environmental surfaces had 10 times greater odds (*p* ≤ 0.05) of having positive employees compared to locations with no positive surfaces.	Environmental surface testing results can be used to inform the need for employee testing
[70]	PPE/Hospital	The risk of COVID-19 infection was more than double among HCWs self-washing their masks compared with the hospital laundry (HR 2.04 (95% CI 1.03 to 4.00); *p* = 0.04). There was no significant difference in infection between HCWs who wore cloth masks washed in the hospital laundry compared with medical masks (*p* = 0.5).	Self-washing cloth masks by hand more than doubles the risk of seasonal respiratory illnesses. Double-layered cloth masks washed in the hospital laundry were as protective as medical masks.
[52]	PPE/Meatpacking	After initiating both universal masking and physical barrier interventions, 8/11 facilities showed a statistically significant reduction in COVID-19 incidence in <10 days. Facilities that only initiated a universal mask policy showed no significant difference before and after the intervention.	Together, universal masking and physical barriers can prevent COVID-19 transmission in meatpacking plants. These interventions should be accompanied by ventilation enhancements and worker education on mask use and adherence.
[51]	Education/Nursing home	Special focus facilities (*n* = 123) started with higher infection rates than control facilities (*n* = 237) but rapidly declined to the same level as the other facilities within a week of starting on-site and virtual infection control consultations. Compliance with cohorting and PPE were associated with large reductions in the weekly infection rate (−50%; *p* = .004; −23%; *p* = .0379) and increased odds of a zero-infection rate ([OR] = 3.0; *p* = .0076; OR = 2.16; *p* = .0003).	Monitored adherence to infection control processes, especially proper wearing of PPE and cohorting, can reduce weekly infections and mortality.
[72]	Changes in work arrangements/Nursing home	Long-term care facilities (LTCF) that organized staff compartmentalization within zones were significantly more likely to avoid a COVID-19 outbreak (OR = 0.19 (0.07–0.48)) as were LTCFs whose staff perceived high-quality implementation of preventive measures (OR = 0.65 (0.43–0.98)).	Staff compartmentalization within zones and high-quality implementation of preventive measures can help prevent COVID-19 outbreaks in LTCFs.
[53]	Changes in work arrangements/Nursing home	Staff working across different care homes (14/27, 52%) had a 3.0-fold (95% CI, 1.9–4.8; *p* < 0.001) higher risk of SARS-CoV-2 positivity than staff working in single care homes (39/227, 17%). Whole-genome sequencing identified distinct clusters of SARS-CoV-2 infection between staff only, including those with minimal resident contact.	Staff should be encouraged and incentivized to work in single care homes and movement of staff across multiple care homes should be limited. Infection control should be extended for all contacts, including those between staff, whilst on the care home premises.

## Data Availability

Data used for meta-analysis is provided in the Supplemental Materials under Supplemental Sheet S1: Meta-analysis data.

## Supplementary Materials

The following are available online at https://www.mdpi.com/article/10.3390/ijerph18157847/s1, Table S1: Quality assessment checklist for included longitudinal studies; Table S2: CHARMS Quality Assessment of Modelling Studies; Sheet S1: Meta-analysis data.
