# Supplementary material for: COVID-19 Prevention and Control Measures in Workplace Settings: A Rapid Review and Meta-Analysis
Source: Int J Environ Res Public Health. 2021 Jul 24;18(15):7847. doi: 10.3390/ijerph18157847 (PMC8345343; doi:10.3390/ijerph18157847)
Supplement: Supplementary file 1 [file ijerph-18-07847-s001.zip › Supplemental Table S1.pdf]

International Journal of
Environmental Research and Public Health

Supplemental Table S1. Quality assessment checklist for included longitudinal studies (n=49).

Author	Design	Experimental (Y/N)	Complete Denominator (Y/N)	PCR Testing Used (Y/N)	Follow Up Time	Overall Score
Lentz et al., 2020	Case-control study	N	Y	N	N	1
Roxby et al., 2020	Case-series Study	N	Y	Y	N	1
Kong et al., 2020	Cohort Study	N	Y	N	Y	2
Miller et al., 2020	Cohort Study	N	Y	N	N	1
Mandić-Rajčević et al., 2020	Epidemiological observational study	N	Y	Y	Y	3
Lipsitz et al., 2020	Longitudinal Cohort Study	N	Y	N	Y	2
Reyné et al., 2020	Observational Retrospective Study	N	Y	Y	N	2
Krone et al., 2021	Observational Study	N	Y	Y	N	2
Wee et al., 2020	Observational Study	N	Y	Y	Y	3
LEI Wee et al., 2020	Observational Study	N	Y	Y	Y	3
Kabesch et al.,2020	Outbreak Investigation Report	N	N	Y	Y	2
Knoll et al., 2020	Outbreak Investigation Report	N	Y	Y	Y	3
MacIntyre et al., 2020	Post-hoc Analysis of a randomized controlled trial	Y	Y	N	Y	3
Dubey et al., 2020	Prospective Cohort Study	N	Y	Y	N	2
Benea et al., 2020	Prospective Cohort Study	N	Y	Y	Y	3
Chang et al., 2020	Prospective Cohort Study	N	Y	Y	Y	3
Chung et al., 2020	Prospective cohort study	N	Y	Y	N	2
Jones et al., 2020	Prospective Cohort Study	N	Y	Y	Y	3
Karmarkar et al., 2020	Prospective Cohort Study	N	Y	Y	Y	3
Mortgat et al., 2020	Prospective Cohort Study	N	Y	Y	N	2
Luigi et al., 2020	Prospective Cohort Study	N	Y	N	Y	2

Author	Design	Experimental (Y/N)	Complete Denominator (Y/N)	PCR Testing Used (Y/N)	Follow Up Time	Overall Score
Menting et al., 2021	Prospective Cohort Study	N	Y	Y	Y	3
Rivett et al.,2020	Prospective Cohort Study	N	Y	Y	Y	3
Mohanty et al., 2020	Prospective Cohort Study	N	Y	N	Y	2
Seidelman et al.,2020	Prospective Cohort Study	N	N	N	Y	1
Squeri et al., 2020	Prospective Cohort Study	N	Y	N	N	1
Suzuki et al., 2021	Prospective Cohort Study	N	Y	N	N	1
Wee et al., 2020	Prospective Cohort Study	N	Y	Y	Y	3
Woon et al., 2020	Prospective Cohort Study	N	Y	Y	N	2
Wang DD et al., 2021	Prospective Cohort Study	N	Y	Y	Y	3
Brehm et al., 2020	Prospective longitudinal seroconversion study	N	Y	Y	Y	3
Cattelan et al., 2020	Prospective Observational Study	N	Y	Y	Y	3
Kumar 2020	Prospective observational study	N	Y	Y	Y	3
Eyre et al., 2020	Prospective Observational Study	N	Y	Y	Y	3
Erber et al., 2020	Prospective seroprevalence study	N	Y	Y	Y	3
Gunawardana et al.,2020	Prospective, longitudinal, observational	N	Y	Y	N	2
Huy et al., 2020	Prospective Descriptive Study	N	Y	Y	N	2
Sanchez et al., 2020	Report	N	Y	Y	Y	3
Brainard et al., 2020	Retrospective Cohort Study	N	Y	N	Y	2
Canetti et al., 2020	Retrospective Cohort Study	N	Y	Y	N	2
Kim et al., 2020	Retrospective Cohort Study	N	Y	Y	N	2
Ladhani et al., 2020	Retrospective Cohort Study	N	Y	Y	N	2
Wang et al., 2020	Retrospective Cohort Study	N	Y	Y	Y	3

**Author**	**Design**	**Experimental (Y/N)**	**Complete Denominator (Y/N)**	**PCR Testing Used (Y/N)**	**Follow Up Time**	**Overall Score**
Herstein et al., 2021	Retrospective Cohort Study	N	Y	N	N	1
Tripathy et al., 2020	Retrospective Cohort Study	N	Y	Y	Y	3
Rolland et al., 2020	Short Term Prospective Study	N	Y	Y	N	2
ChiChen et al., 2020	Surveillance Study	N	Y	Y	Y	3
Porru et al., 2020	Surveillance Study	N	Y	Y	Y	3
