# Supplementary material for: COVID-19 Prevention and Control Measures in Workplace Settings: A Rapid Review and Meta-Analysis
Source: Int J Environ Res Public Health. 2021 Jul 24;18(15):7847. doi: 10.3390/ijerph18157847 (PMC8345343; doi:10.3390/ijerph18157847)
Supplement: Supplementary file 1 [file ijerph-18-07847-s001.zip › Supplemental Table S2.pdf]

International Journal of
Environmental Research and Public Health

MDPI

Supplemental Table S2. CHARMS Quality Assessment of Modelling Studies

Domain	Key items	General	Applicability	Risk of Bias
**Author**	**Grassly et al., 2020**			
**SOURCE OF DATA**	Source of data (e.g., cohort, case-control, randomized trial participants, or registry data)	✔	✔	X
**PARTICIPANTS**	Participant eligibility and recruitment method (e.g., consecutive participants, location, number of centers, setting, inclusion and exclusion criteria)	X	X	X
	Participant description	X	X	X
	Details of treatments received, if relevant	✔	✔	X
	Study dates	X	X	X
**OUTCOME(S) TO BE PREDICTED**	Definition and method for measurement of outcome	✔	✔	X
	Was the same outcome definition (and method for measurement) used in all patients?	✔	✔	X
	Type of outcome (e.g., single or combined endpoints)	✔	X	X
	Was the outcome assessed without knowledge of the candidate predictors (i.e., blinded)?	X	X	X
	Were candidate predictors part of the outcome (e.g., in panel or consensus diagnosis)?	X	X	X
	Time of outcome occurrence or summary of duration of follow-up	X	X	X
**CANDIDATE PREDICTORS (OR INDEX TESTS)**	Number and type of predictors (e.g., demographics, patient history, physical examination, additional testing, disease characteristics)	✔	✔	X
	Definition and method for measurement of candidate predictors	✔	✔	X
	Timing of predictor measurement (e.g., at patient	✔	✔	X

	presentation, at diagnosis, at treatment initiation)			
	Were predictors assessed blinded for outcome, and for each other (if relevant)?	X	X	X
	Handling of predictors in the modelling (e.g., continuous, linear, non-linear transformations or categorised)	X	X	X
**SAMPLE SIZE**	Number of participants and number of outcomes/events	X	X	X
	Number of outcomes/events in relation to the number of candidate predictors (Events Per Variable)	X	X	X
**MISSING DATA**	Number of participants with any missing value (include predictors and outcomes)	X	X	X
	Number of participants with missing data for each predictor	X	X	X
	Handling of missing data (e.g., complete-case analysis, imputation, or other methods)	X	X	X
**MODEL DEVELOPMENT**	Modelling method (e.g., logistic, survival, neural network, or machine learning techniques)	✔	✔	X
	Modelling assumptions satisfied	✔	✔	X
	Method for selection of predictors **for inclusion** in multivariable modelling (e.g., all candidate predictors, pre-selection based on unadjusted association with the outcome)	✔	✔	X
	Method for selection of predictors **during multivariable modelling** (e.g., full model approach, backward or forward selection) and criteria used (e.g., p-value, Akaike Information Criterion)	✔	✔	X
	Shrinkage of predictor weights or regression coefficients (e.g., no shrinkage, uniform shrinkage, penalized estimation)	X	X	X
**MODEL PERFORMANCE**	Calibration (calibration plot, calibration slope, Hosmer-Lemeshow test) and Discrimination (C-statistic, D-statistic, log-rank) measures with confidence inter-	X	X	X

	vals			
	Classification measures (e.g., sensitivity, specificity, predictive values, net reclassification improvement) and whether a-priori cut points were used	✔	X	X
**MODEL EVALUATION**	Method used for testing model performance: development dataset only (random split of data, resampling methods e.g. bootstrap or cross-validation, none) or separate external validation (e.g. temporal, geographical, different setting, different investigators)	✔	✔	X
	In case of poor validation, whether model was adjusted or updated (e.g., intercept recalibrated, predictor effects adjusted, or new predictors added)	X	X	X
**RESULTS**	Final and other multivariable models (e.g., basic, extended, simplified) presented, including predictor weights or regression coefficients, intercept, baseline survival, model performance measures (with standard errors or confidence intervals)	X	X	X
	Any alternative presentation of the final prediction models, e.g., sum score, nomogram, score chart, predictions for specific risk subgroups with performance	✔	✔	X
	Comparison of the distribution of predictors (including missing data) for development and validation datasets	X	X	X
**INTERPRETATION AND DISCUSSION**	Interpretation of presented models (confirmatory, i.e., model useful for practice versus exploratory, i.e., more research needed)	✔	✔	X
	Comparison with other studies, discussion of generalizability, strengths, and limitations.	✔	✔	X

Domain	Key items	General	Applicabil-	Risk

			ity	of Bias
**Author**	Qiu et al., 2020			
**SOURCE OF DATA**	Source of data (e.g., cohort, case-control, randomized trial participants, or registry data)	X	X	X
**PARTICIPANTS**	Participant eligibility and recruitment method (e.g., consecutive participants, location, number of centers, setting, inclusion and exclusion criteria)	X	X	X
	Participant description	X	X	X
	Details of treatments received, if relevant	X	X	X
	Study dates	X	X	X
**OUTCOME(S) TO BE PREDICTED**	Definition and method for measurement of outcome	✔	✔	X
	Was the same outcome definition (and method for measurement) used in all patients?	✔	✔	X
	Type of outcome (e.g., single or combined endpoints)	✔	✔	X
	Was the outcome assessed without knowledge of the candidate predictors (i.e., blinded)?	X	X	X
	Were candidate predictors part of the outcome (e.g., in panel or consensus diagnosis)?	✔	✔	X
	Time of outcome occurrence or summary of duration of follow-up	X	X	X
**CANDIDATE PREDICTORS (OR INDEX TESTS)**	Number and type of predictors (e.g., demographics, patient history, physical examination, additional testing, disease characteristics)	✔	✔	X
	Definition and method for measurement of candidate predictors	✔	✔	X
	Timing of predictor measurement (e.g., at patient presentation, at diagnosis, at treatment initiation)	✔	✔	X
	Were predictors assessed blinded for outcome, and for each other (if relevant)?	X	X	X

	Handling of predictors in the modelling (e.g., continuous, linear, non-linear transformations or categorised)	✔	✔	X
**SAMPLE SIZE**	Number of participants and number of outcomes/events	X	X	X
	Number of outcomes/events in relation to the number of candidate predictors (Events Per Variable)	X	X	X
**MISSING DATA**	Number of participants with any missing value (include predictors and outcomes)	X	X	X
	Number of participants with missing data for each predictor	X	X	X
	Handling of missing data (e.g., complete-case analysis, imputation, or other methods)	X	X	X
**MODEL DEVELOPMENT**	Modelling method (e.g., logistic, survival, neural network, or machine learning techniques)	✔	✔	X
	Modelling assumptions satisfied	✔	✔	X
	Method for selection of predictors **for inclusion** in multivariable modelling (e.g., all candidate predictors, pre-selection based on unadjusted association with the outcome)	✔	✔	X
	Method for selection of predictors **during multivariable modelling** (e.g., full model approach, backward or forward selection) and criteria used (e.g., p-value, Akaike Information Criterion)	✔	✔	X
	Shrinkage of predictor weights or regression coefficients (e.g., no shrinkage, uniform shrinkage, penalized estimation)	X	X	X
**MODEL PERFORMANCE**	Calibration (calibration plot, calibration slope, Hosmer-Lemeshow test) and Discrimination (C-statistic, D-statistic, log-rank) measures with confidence intervals	X	X	X
	Classification measures (e.g., sensitivity, specificity, predictive values, net reclassification improvement)	X	X	X

	and whether a-priori cut points were used			
**MODEL EVALUATION**	Method used for testing model performance: development dataset only (random split of data, resampling methods e.g. bootstrap or cross-validation, none) or separate external validation (e.g. temporal, geographical, different setting, different investigators)	✔	✔	X
	In case of poor validation, whether model was adjusted or updated (e.g., intercept recalibrated, predictor effects adjusted, or new predictors added)	X	X	X
**RESULTS**	Final and other multivariable models (e.g., basic, extended, simplified) presented, including predictor weights or regression coefficients, intercept, baseline survival, model performance measures (with standard errors or confidence intervals)	✔	✔	X
	Any alternative presentation of the final prediction models, e.g., sum score, nomogram, score chart, predictions for specific risk subgroups with performance	✔	✔	X
	Comparison of the distribution of predictors (including missing data) for development and validation datasets	X	X	X
**INTERPRETATION AND DISCUSSION**	Interpretation of presented models (confirmatory, i.e., model useful for practice versus exploratory, i.e., more research needed)	✔	✔	X
	Comparison with other studies, discussion of generalizability, strengths, and limitations.	✔	✔	X

Domain	Key items	General	Applicability	Risk of Bias
**Author**	**Chin et al., 2020**			

**SOURCE OF DATA**	Source of data (e.g., cohort, case-control, randomized trial participants, or registry data)	X	X	X
**PARTICIPANTS**	Participant eligibility and recruitment method (e.g., consecutive participants, location, number of centers, setting, inclusion and exclusion criteria)	✔	✔	X
	Participant description	✔	✔	X
	Details of treatments received, if relevant	X	X	X
	Study dates	X	X	X
**OUTCOME(S) TO BE PREDICTED**	Definition and method for measurement of outcome	✔	✔	X
	Was the same outcome definition (and method for measurement) used in all patients?	✔	✔	X
	Type of outcome (e.g., single or combined endpoints)	✔	✔	X
	Was the outcome assessed without knowledge of the candidate predictors (i.e., blinded)?	X	X	X
	Were candidate predictors part of the outcome (e.g., in panel or consensus diagnosis)?	✔	✔	X
	Time of outcome occurrence or summary of duration of follow-up	✔	✔	X
**CANDIDATE PREDICTORS (OR INDEX TESTS)**	Number and type of predictors (e.g., demographics, patient history, physical examination, additional testing, disease characteristics)	✔	✔	X
	Definition and method for measurement of candidate predictors	✔	✔	X
	Timing of predictor measurement (e.g., at patient presentation, at diagnosis, at treatment initiation)	✔	✔	X
	Were predictors assessed blinded for outcome, and for each other (if relevant)?	X	X	X
	Handling of predictors in the modelling (e.g., continuous, linear, non-linear transformations or categorised)	✔	✔	X
**SAMPLE SIZE**	Number of participants and number of out-	✔	✔	X

	comes/events			
	Number of outcomes/events in relation to the number of candidate predictors (Events Per Variable)	X	X	X
**MISSING DATA**	Number of participants with any missing value (include predictors and outcomes)	X	X	X
	Number of participants with missing data for each predictor	X	X	X
	Handling of missing data (e.g., complete-case analysis, imputation, or other methods)	X	X	X
**MODEL DEVELOPMENT**	Modelling method (e.g., logistic, survival, neural network, or machine learning techniques)	✔	✔	X
	Modelling assumptions satisfied	✔	✔	X
	Method for selection of predictors **for inclusion** in multivariable modelling (e.g., all candidate predictors, pre-selection based on unadjusted association with the outcome)	X	X	X
	Method for selection of predictors **during multivariable modelling** (e.g., full model approach, backward or forward selection) and criteria used (e.g., p-value, Akaike Information Criterion)	X	X	X
	Shrinkage of predictor weights or regression coefficients (e.g., no shrinkage, uniform shrinkage, penalized estimation)	X	X	X
**MODEL PERFORMANCE**	Calibration (calibration plot, calibration slope, Hosmer-Lemeshow test) and Discrimination (C-statistic, D-statistic, log-rank) measures with confidence intervals	✔	✔	X
	Classification measures (e.g., sensitivity, specificity, predictive values, net reclassification improvement) and whether a-priori cut points were used	✔	✔	X
**MODEL**	Method used for testing model performance: development dataset only (random split of data, resampling methods e.g. bootstrap or cross-validation, none) or	X	X	X

**EVALUATION**	separate external validation (e.g. temporal, geographical, different setting, different investigators)			
	In case of poor validation, whether model was adjusted or updated (e.g., intercept recalibrated, predictor effects adjusted, or new predictors added)	X	X	X
**RESULTS**	Final and other multivariable models (e.g., basic, extended, simplified) presented, including predictor weights or regression coefficients, intercept, baseline survival, model performance measures (with standard errors or confidence intervals)	✔	✔	X
	Any alternative presentation of the final prediction models, e.g., sum score, nomogram, score chart, predictions for specific risk subgroups with performance	X	X	X
	Comparison of the distribution of predictors (including missing data) for development and validation datasets	X	X	X
**INTERPRETATION AND DISCUSSION**	Interpretation of presented models (confirmatory, i.e., model useful for practice versus exploratory, i.e., more research needed)	✔	✔	X
	Comparison with other studies, discussion of generalizability, strengths, and limitations.	✔	✔	X

**Domain**	**Key items**	**General**	**Applicability**	**Risk of Bias**
**Author**	Kucharski et al., 2020			
**SOURCE OF DATA**	Source of data (e.g., cohort, case-control, randomized trial participants, or registry data)	✔	✔	X
**PARTICIPANTS**	Participant eligibility and recruitment method (e.g., consecutive participants, location, number of centers,	✔	✔	X

	setting, inclusion and exclusion criteria)			
	Participant description	✔	✔	X
	Details of treatments received, if relevant	✔	✔	X
	Study dates	X	X	X
**OUTCOME(S) TO BE PREDICTED**	Definition and method for measurement of outcome	✔	✔	X
	Was the same outcome definition (and method for measurement) used in all patients?	✔	✔	X
	Type of outcome (e.g., single or combined endpoints)	✔	✔	X
	Was the outcome assessed without knowledge of the candidate predictors (i.e., blinded)?	X	X	X
	Were candidate predictors part of the outcome (e.g., in panel or consensus diagnosis)?	✔	✔	X
	Time of outcome occurrence or summary of duration of follow-up	X	X	X
**CANDIDATE PREDICTORS (OR INDEX TESTS)**	Number and type of predictors (e.g., demographics, patient history, physical examination, additional testing, disease characteristics)	✔	✔	X
	Definition and method for measurement of candidate predictors	✔	✔	X
	Timing of predictor measurement (e.g., at patient presentation, at diagnosis, at treatment initiation)	X	X	X
	Were predictors assessed blinded for outcome, and for each other (if relevant)?	X	X	X
	Handling of predictors in the modelling (e.g., continuous, linear, non-linear transformations or categorised)	✔	✔	X
**SAMPLE SIZE**	Number of participants and number of outcomes/events	✔	✔	X
	Number of outcomes/events in relation to the number of candidate predictors (Events Per Variable)	✔	✔	X
**MISSING DATA**	Number of participants with any missing value (in-	X	X	X

	clude predictors and outcomes)			
	Number of participants with missing data for each predictor	X	X	X
	Handling of missing data (e.g., complete-case analysis, imputation, or other methods)	X	X	X
**MODEL DEVELOPMENT**	Modelling method (e.g., logistic, survival, neural network, or machine learning techniques)	✔	✔	X
	Modelling assumptions satisfied	✔	✔	X
	Method for selection of predictors **for inclusion** in multivariable modelling (e.g., all candidate predictors, pre-selection based on unadjusted association with the outcome)	X	X	X
	Method for selection of predictors **during multivariable modelling** (e.g., full model approach, backward or forward selection) and criteria used (e.g., p-value, Akaike Information Criterion)	X	X	X
	Shrinkage of predictor weights or regression coefficients (e.g., no shrinkage, uniform shrinkage, penalized estimation)	X	X	X
**MODEL PERFORMANCE**	Calibration (calibration plot, calibration slope, Hosmer-Lemeshow test) and Discrimination (C-statistic, D-statistic, log-rank) measures with confidence intervals	X	X	X
	Classification measures (e.g., sensitivity, specificity, predictive values, net reclassification improvement) and whether a-priori cut points were used	X	X	X
**MODEL EVALUATION**	Method used for testing model performance: development dataset only (random split of data, resampling methods e.g. bootstrap or cross-validation, none) or separate external validation (e.g. temporal, geographical, different setting, different investigators)	X	X	X

	In case of poor validation, whether model was adjusted or updated (e.g., intercept recalibrated, predictor effects adjusted, or new predictors added)	✔	✔	X
**RESULTS**	Final and other multivariable models (e.g., basic, extended, simplified) presented, including predictor weights or regression coefficients, intercept, baseline survival, model performance measures (with standard errors or confidence intervals)	✔	✔	X
	Any alternative presentation of the final prediction models, e.g., sum score, nomogram, score chart, predictions for specific risk subgroups with performance	✔	✔	X
	Comparison of the distribution of predictors (including missing data) for development and validation datasets	X	X	X
**INTERPRETATION AND DISCUSSION**	Interpretation of presented models (confirmatory, i.e., model useful for practice versus exploratory, i.e., more research needed)	✔	✔	X
	Comparison with other studies, discussion of generalizability, strengths, and limitations.	✔	✔	X

**Domain**	**Key items**	**General**	**Applicability**	**Risk of Bias**
**Author**	**Razzak et al., 2020**			
**SOURCE OF DATA**	Source of data (e.g., cohort, case-control, randomized trial participants, or registry data)	✔	✔	X
**PARTICIPANTS**	Participant eligibility and recruitment method (e.g., consecutive participants, location, number of centers, setting, inclusion and exclusion criteria)	✔	✔	X
	Participant description	✔	✔	X
	Details of treatments received, if relevant	X	X	X

	Study dates	✔	✔	X
**OUTCOME(S) TO BE PREDICTED**	Definition and method for measurement of outcome	✔	✔	X
	Was the same outcome definition (and method for measurement) used in all patients?	✔	✔	X
	Type of outcome (e.g., single or combined endpoints)	✔	✔	X
	Was the outcome assessed without knowledge of the candidate predictors (i.e., blinded)?	✔	✔	X
	Were candidate predictors part of the outcome (e.g., in panel or consensus diagnosis)?	X	X	X
	Time of outcome occurrence or summary of duration of follow-up	X	X	X
**CANDIDATE PREDICTORS (OR INDEX TESTS)**	Number and type of predictors (e.g., demographics, patient history, physical examination, additional testing, disease characteristics)	✔	✔	X
	Definition and method for measurement of candidate predictors	✔	✔	X
	Timing of predictor measurement (e.g., at patient presentation, at diagnosis, at treatment initiation)	✔	✔	X
	Were predictors assessed blinded for outcome, and for each other (if relevant)?	X	X	X
	Handling of predictors in the modelling (e.g., continuous, linear, non-linear transformations or categorised)	✔	✔	X
**SAMPLE SIZE**	Number of participants and number of outcomes/events	✔	✔	X
	Number of outcomes/events in relation to the number of candidate predictors (Events Per Variable)	✔	✔	X
**MISSING DATA**	Number of participants with any missing value (include predictors and outcomes)	X	X	X
	Number of participants with missing data for each predictor	X	X	X

	Handling of missing data (e.g., complete-case analysis, imputation, or other methods)	X	X	X
**MODEL DEVELOPMENT**	Modelling method (e.g., logistic, survival, neural network, or machine learning techniques)	✔	✔	X
	Modelling assumptions satisfied	✔	✔	X
	Method for selection of predictors **for inclusion** in multivariable modelling (e.g., all candidate predictors, pre-selection based on unadjusted association with the outcome)	✔	✔	X
	Method for selection of predictors **during multivariable modelling** (e.g., full model approach, backward or forward selection) and criteria used (e.g., p-value, Akaike Information Criterion)	✔	✔	X
	Shrinkage of predictor weights or regression coefficients (e.g., no shrinkage, uniform shrinkage, penalized estimation)	✔	✔	X
**MODEL PERFORMANCE**	Calibration (calibration plot, calibration slope, Hosmer-Lemeshow test) and Discrimination (C-statistic, D-statistic, log-rank) measures with confidence intervals	✔	✔	X
	Classification measures (e.g., sensitivity, specificity, predictive values, net reclassification improvement) and whether a-priori cut points were used	X	X	X
**MODEL EVALUATION**	Method used for testing model performance: development dataset only (random split of data, resampling methods e.g. bootstrap or cross-validation, none) or separate external validation (e.g. temporal, geographical, different setting, different investigators)	✔	✔	X
	In case of poor validation, whether model was adjusted or updated (e.g., intercept recalibrated, predictor effects adjusted, or new predictors added)	✔	✔	X

**RESULTS**	Final and other multivariable models (e.g., basic, extended, simplified) presented, including predictor weights or regression coefficients, intercept, baseline survival, model performance measures (with standard errors or confidence intervals)	✔	✔	X
	Any alternative presentation of the final prediction models, e.g., sum score, nomogram, score chart, predictions for specific risk subgroups with performance	X	X	X
	Comparison of the distribution of predictors (including missing data) for development and validation datasets	X	X	X
**INTERPRETATION AND DISCUSSION**	Interpretation of presented models (confirmatory, i.e., model useful for practice versus exploratory, i.e., more research needed)	✔	✔	X
	Comparison with other studies, discussion of generalizability, strengths, and limitations.	✔	✔	X

**Domain**	**Key items**	**General**	**Applicability**	**Risk of Bias**
**Author**	**McDermott et al., 2020**			
**SOURCE OF DATA**	Source of data (e.g., cohort, case-control, randomized trial participants, or registry data)	✔	✔	X
**PARTICIPANTS**	Participant eligibility and recruitment method (e.g., consecutive participants, location, number of centers, setting, inclusion and exclusion criteria)	X	X	X
	Participant description	X	X	X
	Details of treatments received, if relevant	X	X	X
	Study dates	X	X	X
**OUTCOME(S) TO BE PREDICTED**	Definition and method for measurement of outcome	✔	✔	X
	Was the same outcome definition (and method for	✔	✔	X

	measurement) used in all patients?			
	Type of outcome (e.g., single or combined endpoints)	✔	✔	X
	Was the outcome assessed without knowledge of the candidate predictors (i.e., blinded)?	X	X	X
	Were candidate predictors part of the outcome (e.g., in panel or consensus diagnosis)?	X	X	X
	Time of outcome occurrence or summary of duration of follow-up	X	X	X
**CANDIDATE PREDICTORS (OR INDEX TESTS)**	Number and type of predictors (e.g., demographics, patient history, physical examination, additional testing, disease characteristics)	X	X	X
	Definition and method for measurement of candidate predictors	✔	✔	X
	Timing of predictor measurement (e.g., at patient presentation, at diagnosis, at treatment initiation)	X	X	X
	Were predictors assessed blinded for outcome, and for each other (if relevant)?	X	X	X
	Handling of predictors in the modelling (e.g., continuous, linear, non-linear transformations or categorised)	✔	✔	X
**SAMPLE SIZE**	Number of participants and number of outcomes/events	X	X	X
	Number of outcomes/events in relation to the number of candidate predictors (Events Per Variable)	X	X	X
**MISSING DATA**	Number of participants with any missing value (include predictors and outcomes)	X	X	X
	Number of participants with missing data for each predictor	X	X	X
	Handling of missing data (e.g., complete-case analysis, imputation, or other methods)	X	X	X
**MODEL**	Modelling method (e.g., logistic, survival, neural network, or machine learning techniques)	✔	✔	X

**DEVELOPMENT**	Modelling assumptions satisfied	✔	X	X
	Method for selection of predictors **for inclusion** in multivariable modelling (e.g., all candidate predictors, pre-selection based on unadjusted association with the outcome)	✔	✔	X
	Method for selection of predictors **during multivariable modelling** (e.g., full model approach, backward or forward selection) and criteria used (e.g., p-value, Akaike Information Criterion)	X	X	X
	Shrinkage of predictor weights or regression coefficients (e.g., no shrinkage, uniform shrinkage, penalized estimation)	X	X	X
**MODEL PERFORMANCE**	Calibration (calibration plot, calibration slope, Hosmer-Lemeshow test) and Discrimination (C-statistic, D-statistic, log-rank) measures with confidence intervals	✔	✔	X
	Classification measures (e.g., sensitivity, specificity, predictive values, net reclassification improvement) and whether a-priori cut points were used	X	X	X
**MODEL EVALUATION**	Method used for testing model performance: development dataset only (random split of data, resampling methods e.g. bootstrap or cross-validation, none) or separate external validation (e.g. temporal, geographical, different setting, different investigators)	✔	✔	X
	In case of poor validation, whether model was adjusted or updated (e.g., intercept recalibrated, predictor effects adjusted, or new predictors added)	X	X	X
**RESULTS**	Final and other multivariable models (e.g., basic, extended, simplified) presented, including predictor weights or regression coefficients, intercept, baseline survival, model performance measures (with standard errors or confidence intervals)	X	X	X

	Any alternative presentation of the final prediction models, e.g., sum score, nomogram, score chart, predictions for specific risk subgroups with performance	✔	✔	X
	Comparison of the distribution of predictors (including missing data) for development and validation datasets	X	X	X
**INTERPRETATION AND DISCUSSION**	Interpretation of presented models (confirmatory, i.e., model useful for practice versus exploratory, i.e., more research needed)	✔	✔	X
	Comparison with other studies, discussion of generalizability, strengths, and limitations.	✔	✔	X

**Domain**	**Key items**	**General**	**Applicability**	**Risk of Bias**
**Author**	**See et al., 2020**			
**SOURCE OF DATA**	Source of data (e.g., cohort, case-control, randomized trial participants, or registry data)	X	X	X
**PARTICIPANTS**	Participant eligibility and recruitment method (e.g., consecutive participants, location, number of centers, setting, inclusion and exclusion criteria)	X	X	X
	Participant description	✔	✔	X
	Details of treatments received, if relevant	✔	✔	X
	Study dates	X	X	X
**OUTCOME(S) TO BE PREDICTED**	Definition and method for measurement of outcome	✔	✔	X
	Was the same outcome definition (and method for measurement) used in all patients?	X	X	X
	Type of outcome (e.g., single or combined endpoints)	X	X	X
	Was the outcome assessed without knowledge of the	X	X	X

	candidate predictors (i.e., blinded)?			
	Were candidate predictors part of the outcome (e.g., in panel or consensus diagnosis)?	X	X	X
	Time of outcome occurrence or summary of duration of follow-up	X	X	X
**CANDIDATE PREDICTORS (OR INDEX TESTS)**	Number and type of predictors (e.g., demographics, patient history, physical examination, additional testing, disease characteristics)	✔	✔	X
	Definition and method for measurement of candidate predictors	✔	✔	X
	Timing of predictor measurement (e.g., at patient presentation, at diagnosis, at treatment initiation)	X	X	X
	Were predictors assessed blinded for outcome, and for each other (if relevant)?	X	X	X
	Handling of predictors in the modelling (e.g., continuous, linear, non-linear transformations or categorised)	X	X	X
**SAMPLE SIZE**	Number of participants and number of outcomes/events	X	X	X
	Number of outcomes/events in relation to the number of candidate predictors (Events Per Variable)	X	X	X
**MISSING DATA**	Number of participants with any missing value (include predictors and outcomes)	X	X	X
	Number of participants with missing data for each predictor	X	X	X
	Handling of missing data (e.g., complete-case analysis, imputation, or other methods)	X	X	X
**MODEL DEVELOPMENT**	Modelling method (e.g., logistic, survival, neural network, or machine learning techniques)	✔	✔	X
	Modelling assumptions satisfied	X	X	X
	Method for selection of predictors **for inclusion** in multivariable modelling (e.g., all candidate predictors,	X	X	X

	pre-selection based on unadjusted association with the outcome)			
	Method for selection of predictors **during multivariable modelling** (e.g., full model approach, backward or forward selection) and criteria used (e.g., p-value, Akaike Information Criterion)	X	X	X
	Shrinkage of predictor weights or regression coefficients (e.g., no shrinkage, uniform shrinkage, penalized estimation)	X	X	X
**MODEL PERFORMANCE**	Calibration (calibration plot, calibration slope, Hosmer-Lemeshow test) and Discrimination (C-statistic, D-statistic, log-rank) measures with confidence intervals	X	X	X
	Classification measures (e.g., sensitivity, specificity, predictive values, net reclassification improvement) and whether a-priori cut points were used	✔	✔	X
**MODEL EVALUATION**	Method used for testing model performance: development dataset only (random split of data, resampling methods e.g. bootstrap or cross-validation, none) or separate external validation (e.g. temporal, geographical, different setting, different investigators)	X	X	X
	In case of poor validation, whether model was adjusted or updated (e.g., intercept recalibrated, predictor effects adjusted, or new predictors added)	X	X	X
**RESULTS**	Final and other multivariable models (e.g., basic, extended, simplified) presented, including predictor weights or regression coefficients, intercept, baseline survival, model performance measures (with standard errors or confidence intervals)	X	X	X
	Any alternative presentation of the final prediction models, e.g., sum score, nomogram, score chart, predictions for specific risk subgroups with performance	✔	✔	X

	Comparison of the distribution of predictors (including missing data) for development and validation datasets	X	X	X
**INTERPRETATION AND DISCUSSION**	Interpretation of presented models (confirmatory, i.e., model useful for practice versus exploratory, i.e., more research needed)	✔	✔	X
	Comparison with other studies, discussion of generalizability, strengths, and limitations.	✔	✔	X
